# A Neuro-Mechanical Model Explaining the Physiological Role of Fast and Slow Muscle Fibres at Stop and Start of Stepping of an Insect Leg

**DOI:** 10.1371/journal.pone.0078246

**Published:** 2013-11-22

**Authors:** Tibor Istvan Toth, Martyna Grabowska, Joachim Schmidt, Ansgar Büschges, Silvia Daun-Gruhn

**Affiliations:** 1 Emmy Noether Research Group of Computational Biology, Department of Animal Physiology, University of Cologne, Cologne, Germany; 2 Department of Animal Physiology, University of Cologne, Cologne, Germany; Mount Sinai School of Medicine, United States of America

## Abstract

Stop and start of stepping are two basic actions of the musculo-skeletal system of a leg. Although they are basic phenomena, they require the coordinated activities of the leg muscles. However, little is known of the details of how these activities are generated by the interactions between the local neuronal networks controlling the fast and slow muscle fibres at the individual leg joints. In the present work, we aim at uncovering some of those details using a suitable neuro-mechanical model. It is an extension of the model in the accompanying paper and now includes all three antagonistic muscle pairs of the main joints of an insect leg, together with their dedicated neuronal control, as well as common inhibitory motoneurons and the residual stiffness of the slow muscles. This model enabled us to study putative processes of intra-leg coordination during stop and start of stepping. We also made use of the effects of sensory signals encoding the position and velocity of the leg joints. Where experimental observations are available, the corresponding simulation results are in good agreement with them. Our model makes detailed predictions as to the coordination processes of the individual muscle systems both at stop and start of stepping. In particular, it reveals a possible role of the slow muscle fibres at stop in accelerating the convergence of the leg to its steady-state position. These findings lend our model physiological relevance and can therefore be used to elucidate details of the stop and start of stepping in insects, and perhaps in other animals, too.

## Introduction

When legged animals stop or start stepping, a transition between posture and locomotion takes place. Thus stop and start of stepping are essential basic actions of the musculo-skeletal system of a leg. There is experimental evidence [1] that a leg does not stop randomly during its step cycle. Rather it stops or starts stepping in a systematic way depending on its position within the stepping cycle [1]. It is therefore reasonable to assume that both processes require coordinated actions of the leg muscles. This is presumably achieved by the interactions of the local neuronal networks that control the activity of the leg muscles. For a deeper understanding of the underlying neurophysiological mechanisms of locomotion in insects, it is thus quite important to study and analyze its elementary processes such as stop and start of stepping. This may open up the way for tackling more complex processes of walking in various conditions.

To be specific, in the stick insect, 3 pairs of antagonistic muscles play the major part in locomotion: the m. protractor and retractor coxae at the thorax-coxa (ThC) joint, the m. levator and depressor trochanteris at the coxa-trochanter (CTr) joint, and the m. flexor and extensor tibiae at the femur-tibia (FTi) joint. The coordination of their activity within a leg is achieved by means of proprioceptive sensory signals. They report load or position, or position and (angular) velocity to the nervous system. The load signals are generated in the campaniform sensilla (CS) [2], the position signals in specialized hairfields [3]–[5], and the position and (angular) velocity signals in the chordotonal organs [6], [7]. Chordotonal organs are present in other insects, as well [8], [9]. Their sensory signals are conveyed between the local neuronal networks controlling the activity of the muscle pairs. On the efferent side, one can distinguish between slow and fast muscle fibres constituting each of the above muscles according to their contraction kinetics [10], [11], or histochemical properties [12]. [10] and [11] showed that the two types are anatomically separated in the extensor tibiae muscle of the stick insect but more importantly that they also have different physiological function: fast muscle fibres are active during stepping, only, whereas slow muscle fibres are responsible for maintaining the static position (posture) of the stick insect. Since [12], in a recent work, showed the existence of slow and fast fibres in the other, aforementioned muscles, too, it seems reasonable to assume that they have analogous function in those muscles, as well.

The question now arises whether and how the neuro-muscular system just described can bring about the stop and start of stepping of an insect leg. One suitable way to try to answer this question is to use appropriate mathematical models. In the accompanying paper [34], we presented a neuro-mechanical model that included slow and fast muscle fibres and their dedicated controlling neuronal networks. In this paper, we apply an extended version of this model in an attempt to unveil and elucidate the details of the stopping and starting of stepping. We have thus extended the model in [34] to include four important new properties: i) all six muscle types have both slow and fast fibres; ii) the slow muscle fibres possess residual stiffness, and iii) are controlled by the activity of the common inhibitor motoneuron CI1 (for the flexor tibiae muscle CI2 and CI3); iv) the effects of the position and (angular) velocity sensory signals are implemented. As a result, we can suggest neuro-mechanical mechanisms that might exist in insects at stop and start of stepping. More generally, we hope to have helped gain a deeper understanding of elementary mechanisms of locomotion in insects, and perhaps in other animals, too.

## Methods

### The model comprising all three neuro-muscular systems

**Figure 1:**
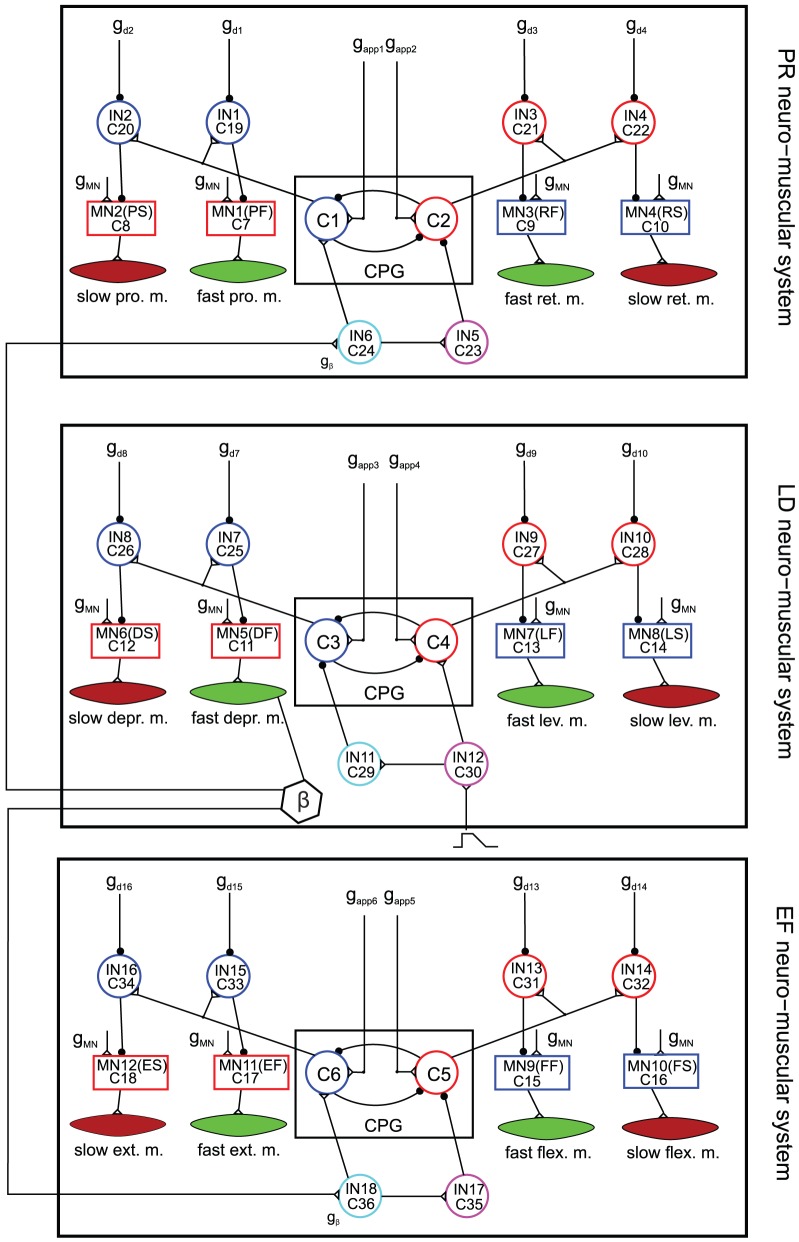
The extended model comprising all three neuro-muscular systems equipped with fast and slow muscle fibres. The **protractor-retractor** (PR) neuro-muscular system consists of a central pattern generator: CPG, slow and fast protractor and retractor muscles as indicated (slow pro. m. etc.), the corresponding motoneurons: MN(PS) etc., 4 inhibitory interneurons (IN1–IN4) connecting the CPG to the motoneurons, and two additional interneurons (IN5–IN6), which convey neuronal signals to the CPG from sense organs of other joints of the same leg, or possibly of other legs. 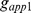, 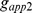 are conductances of the driving currents to C1 and C2, respectively. 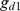
–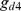 are conductances of the inhibitory currents to IN1–IN4, respectively. 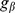 is the conductance of the sensory input current from the levator-depressor muscle system. The **levator-depressor** (LD) neuro-muscular system consists of a central pattern generator: CPG, slow and fast levator and depressor muscles as indicated (slow depr. m. etc.), the corresponding motoneurons: MN(DS) etc., 4 inhibitory interneurons (IN7–IN10) connecting the CPG to the motoneurons, and two additional interneurons (IN11–IN12), which convey neuronal signals to the CPG from peripheral sense organs of the leg (ramp symbol). 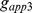, 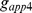 are conductances of the driving currents to C3 and C4, respectively. 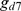
–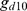 are conductances of the inhibitory currents to IN7–IN10, respectively. The **extensor-flexor** (EF) neuro-muscular system consists of a central pattern generator: CPG, slow and fast extensor and flexor muscles as indicated (slow ext. m. etc.), the corresponding motoneurons: MN(ES) etc., 4 inhibitory interneurons (IN13–IN16) connecting the CPG to the motoneurons, and two additional interneurons (IN17–IN18), which convey neuronal signals to the CPG from sense organs of other joints of the same leg, or possibly of other legs. 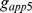, 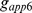 are conductances of the driving currents to C5 and C6, respectively. 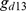
–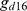 are conductances of the inhibitory currents to IN13–IN16, respectively. 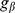 is the conductance of the sensory input current from the levator-depressor muscle system. 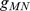 is the conductance of the common (central) input current to all motoneurons. Empty triangles are excitatory synapses; filled black circles on neurons are inhibitory synapses. The tiny black circles on synaptic paths are branching points. The three neuro-muscular systems have been put in frames with reference to Fig. 7 for the sake of their easier identification in that figure.

The model introduced in this paper is an extension of the models in [13] and the accompanying paper [34]. Fig. 1 shows the network with all three neuro-muscular systems. Each of them is now equipped with slow muscles, too, and with motoneurons (MNs) that innervate the slow muscles (in short slow MNs), as well as with the corresponding interneurons (INs). The three systems are coupled via position and load signals [2] represented by the levation angle 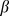 (hexagon with 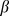 in Fig. 1). If 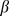 exceeds, or falls below, a critical value (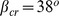 for the protractor-retractor system and 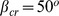 for the extensor-flexor system), it will initiate a new (swing or stance) phase of a stepping cycle. For a more detailed explanation, see [13].

The activation kinetics of a muscle fibre during a contraction initiated by the excitation of its MN determine its type. Thus fast muscle fibres have fast activation kinetics and slow fibres much slower ones compared to those of the fast muscle fibres. The slow kinetics of the slow muscle fibres are therefore characterized by small rate constants, which apply during an incoming action potential. The specific values of the activation rate constants of the fast muscle fibres are listed in Table 1 for each muscle type. These values were chosen in earlier versions of the model [13], [14] such as to fit the movements of the femur and the tibia during the swing and the stance phase of the stepping leg as seen in the experiments [15].

**Table 1:** Activation rate constants of the different fast muscle types.

Muscle type	activation rate constant (1/ms)	
	stance phase	swing phase
Protractor	2.01	0.81
Retractor	5.01	0.71
Levator	15.01	8.01
Depressor	5.01	5.01
Extensor	5.01	5.01
Flexor	8.01	8.01

The values of the activation rate constants of the corresponding slow muscle fibres were set to be 100 times smaller. The relaxation rate constants (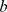 values) were chosen to be identical in both muscle types (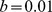 ms^−1^ for all muscle types). Details of the properties of the neuron and muscle models and the neuro-muscular coupling can be found in [14] and in the accompanying paper [34].

**Figure 2:**
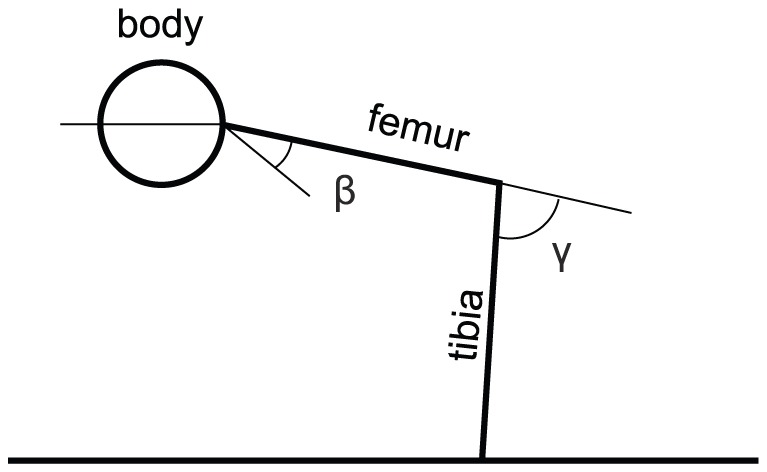
Illustration of the standing position of the middle leg of the stick insect as a projection into the vertical plane. 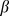
: levation angle 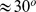, 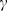: flexion angle 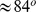. Note that the reference axis of the angle 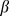 is not horizontal because the longitudinal axis of the coxa is tilted from the horizontal direction by an angle of 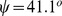 [22].

However, the elastic properties of the slow muscle fibres differ substantially from those of the fast ones. All types of the slow fibres are assumed to have a positive residual stiffness, while the fast ones are not. Formally that means that the actual value, 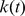, of the stiffness (spring constant) of the *slow* muscles in the absence of an action potential is now calculated as
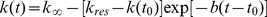
(1)(cf. eqns 1–2 in the accompanying paper [34]). Here is 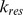, the actual value of the residual stiffness. This value can be affected by the activity of the CI1 (and CI2-CI3) (see below). The residual stiffness ensures that static positions of the stick insect are maintained over a longer period of time with virtually no driving activity of the motoneurons innervating these fibres [16]. The value of the residual stiffness is controlled by the activity of common inhibitory MNs. The common inhibitory MN CI1 innervates slow fibres of five of the six muscles named above. (The slow flexor tibiae muscle is innervated by CI2 and CI3.) [17] performed experiments on the locust and showed that the residual stiffness of the slow muscles is abolished during locomotion (stepping) by the activity of CI1. He suggested that the main physiological role of CI1 (and of the synchronously active CI2 and CI3 in the m. flexor tibiae [18]) is to ensure fast movements of the limbs, especially during the swing phase (e.g. during protraction in the protractor-retractor muscle system). Similar results were obtained in the crab [19] and in the cockroach [20], [21]. We implemented the residual stiffness of the slow muscle fibres in accordance with these findings and hypotheses. Table 2 lists its value for each muscle type. The values in Table 2 were chosen such that the stationary position, i.e. the angles 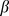 and 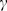 at the coxa-trochanter and the femur-tibia joint, respectively, be in the range of angles measured in the stick insect in its standing resting position (Fig. 2). Thus, 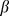 in a small range around 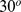 (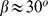), and 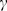 in the range of 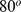 to 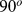 (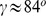) [22]. The position of the femur in the horizontal plane is determined by the angle 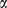. Its stationary value was set to 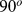, and the values of the residual spring constants of the slow protractor and retractor muscles were calculated accordingly (Table 2). These “basic” stationary values can, of course, be modified by making use of the recruitment properties of the model (cf. Results and the accompanying paper [34]).

**Table 2:** Residual values of the spring constants 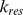 of the different slow muscle types.

Muscle type	*k_res_*. (mN/mm^2^)
Protractor	15.0
Retractor	25.5
Levator	10.0
Depressor	8.8
Extensor	34.0
Flexor	4.3

In the present version of the model, we implemented the function of CI1 implicitly. We did not model the mechanism itself that produces the changes in the residual stiffness of the slow muscle fibres during activity of CI1 but the effect, i.e. the changes, only. Hence, the residual values of the spring constants assume their stationary (nonzero) values during inhibition of the innervating CI1. During stepping, when CI1 is active, the residual values vanish in the swing phase and are small in the stance phase of the stepping. To be more specific, CI1 affects the actual value of the residual stiffness 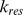 of a slow muscle in the following way:
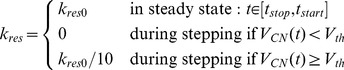
(2)In this eqn, 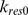 is the value of the residual stiffness 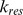 which it assumes during steady state. Furthermore, 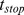 is the onset of the stop command, and 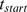 is the onset of the command for re-start. 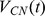, with 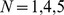, denotes the actual value of the membrane potential of the ‘retractor’ (C1), ‘depressor’ (C4) and ‘extensor’ (C5) CPG neurons' (cf. Fig. 1). 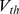 is a threshold value (set to 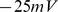 in the simulations). It is used to decide whether the CPGs are in phases corresponding to the swing phase of the stepping cycle, in particular, whether the protractor-retractor CPG is in the protraction phase. That is, if the neurons CN, 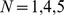 are sufficiently hyperpolarized, the leg is in the swing (protraction) phase. The third condition in eqn 2 means that the stepping movement is in the stance phase. Obviously, during stepping: 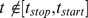. The lack of residual stiffness in the swing phase increases the speed of the leg movement, in accordance with the experimental findings [17], [19], [21]. In the model, we lumped together CI1, which innervates five of the six muscle types, CI2 and CI3, which innervate the flexor tibiae muscle. This simplification is not likely to cause noticeable errors in the simulations, since, as experiments showed, the activities of CI1, CI2 and CI3 are synchronous with good approximation [18], [21].

The various sensory, in particular position (angle) and (angular) velocity, signals were also implicitly modelled. The sense organs that produce such signals do exist for each joint of each leg in insects [6], [8]; for a survey, see [7]. In the model, these signals were treated as physical quantities and were not encoded in neuronal signals. Their effects were therefore implemented as abstract logical decisions or operations (see Results below). Nevertheless, we have endeavoured to make these effects and functions physiologically viable by providing a putative neuronal network that, at least qualitatively, is capable of reproducing them (cf. Discussion).

### A technical note on the usage of muscle fibre recruitment in the model

If the recruitment levels of the slow muscle fibres and the contraction forces in the recruited fibres of a pair of antagonistic muscles are given, they determine a unique stationary position of the leg joint (cf. the accompanying paper [34]). It is however more practical to define the stationary angle (e.g. the horizontal position of the femur by the angle 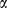) and to determine the recruitment levels in the appropriate muscles (e.g. in the protractor and retractor muscles).

Here is a short description of the calculation of the angle 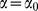, which yields the horizontal resting position of the femur. At the stationary angle 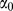, we have [14], [34]

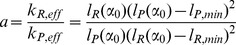
(3)where 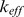 (omitting here and in the subsequent formulae the subscripts indicating the specific muscle type for the sake of simplicity) is the so-called effective spring constant, which is the value corrected for the actual level of recruitment 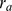; 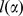 is the muscle length at the angle 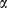, 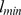 denotes the minimal length of the corresponding muscle (at which the muscle is completely relaxed). Now, 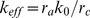, where 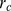 is the control (or reference) recruitment level, and 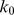 is computed in the model for the recruitment level 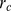 in the first place (cf. the accompanying paper [34]). In the stationary state, we have 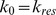, the latter being the residual value of the spring constant 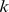. Hence,
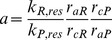
(4)according to [34]. From this eqn, we obtain
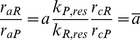
(5)If 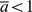, we set 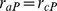 and 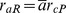. If 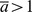, then analogously 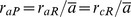. (At 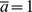 trivially 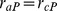 and 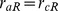.)

When the stick insect is standing in the usual (normal) position (cf. Fig. 2), then no calculation of the recruitment levels is needed for the other two muscle pairs. The stationary values of the two corresponding angles 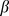 and 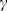 are namely predefined by the normal standing position. That is, we have 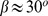 and 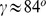. It is, of course, not difficult to apply the reasoning we have just used for the protractor-retractor system to the other two neuro-muscular systems, if required.

## Results

In the model, we assumed that sensory signals reflecting the positions (angles) and the (angular) velocities of the joints of the middle leg were used to coordinate the movements of the femur and tibia both at stopping and starting the locomotion (stepping). According to our hypothesis, both processes were triggered by mutually exclusive command signals of central origin. First, we deal with the stop of stepping.

### Stop of stepping

We recall the definitions of the angles 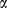, 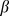 and 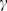 [13], [14]: 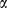 increases during retraction, i.e. moving the femur backward in the horizontal plane; 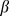 increases during levation, i.e. lifting the femur off the ground; and 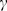 increases during flexion, i.e. moving the tibia towards the body in the vertical plane.

With these conventions in mind, we assumed that the stopping process must start with 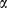 and 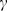 both in the decreasing phase, i.e. with protraction and extension of the femur and the tibia, respectively. This constraint ensured that the leg movement never finished with a protraction in the stance phase. Such a situation was never observed in experiments ([1], and Grabowska, unpublished data). It is however self evident that the last phase before steady state is always a stance phase [1], since ground contact must have been established before, or at, a complete still stand. This means that the central command signal to stop became effective only after 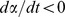 and 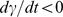 had been fulfilled. More formally, we have
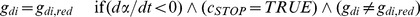
(6)

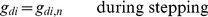
(7)for 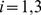 (cf. Fig. 1). In these eqns, 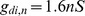 is the value of 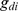 during stepping (‘normal’ value), and 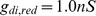 is its value during steady state (‘reduced’ value). 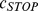 is the boolean variable for the stop command, and ‘
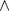
’ is the boolean ‘and’ operator. The last inequality condition in eqn 6 means that the switch in the value of 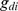 occurs only once while the stop command is on. If we set 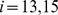, substitute 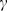 for 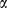 in eqn 6, we obtain a completely analogous condition for stopping the fast MNs of the extensor-flexor neuro-muscular system. Once these conditions were met, the activity of the fast MNs was inhibited via the inhibitory INs. Consequently, the fast muscle fibres in all four muscles: protractor, retractor and extensor, flexor relaxed and did not exert any torque on the thorax-coxa and femur-tibia joints, respectively. As far as the corresponding slow MNs are concerned, their activity was enhanced upon onset of the stop command due to the inhibition of their inhibitory INs: Again, expressing this in form of equations, we have
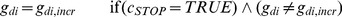
(8)

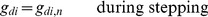
(9)for 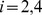 and 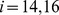 in the protractor-retractor and the extensor-flexor neuro-muscular system, respectively. In eqn 8, 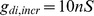 is the enhanced conductance of the (central) inhibitory current to the IN (cf. Fig. 1). The disinhibition of the corresponding slow MNs resulted, of course, in stronger activity of the slow muscles innervated by these MNs.

The negative angular velocities above were preconditions for establishing and maintaining permanent ground contact by the levator-depressor neuro-muscular system. However, they had to be complemented by one condition on the angular signal 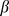, which had to reach the value at ground contact (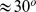) in order that both *fast and slow* levator and depressor MNs became inhibited. In equation form, these conditions read
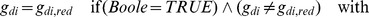


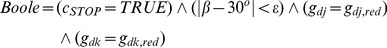
(10)

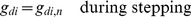
(11)for 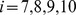, 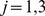, and 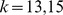; 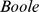 is a boolean variable, and 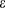 is a sufficiently small value (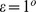). Thus, once the conditions in eqn 10 were fulfilled, a permanent ground contact was established. The (middle) leg came to a complete rest, having performed a retraction in the stance phase, while approaching the stationary angle 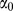. The activity of slow muscle fibres of both the protractor-retractor and the extensor-flexor system was only stopped when the angles 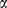 and 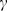 were close enough (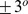) to their stationary values (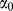 and 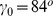) and the angular velocities 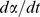 and 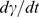 were nearly zero. These conditions, like the previous ones, can also be expressed as equations.
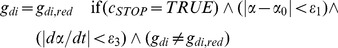
(12)

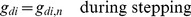
(13)for 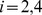 (cf. Fig. 1). Here, 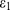 and 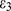 are small values (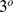 and 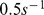, respectively). Setting 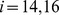 and substituting 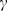 for 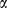, we obtain analogous conditions for stopping the activity of the slow MNs of the extensor-flexor neuro-muscular system.

**Figure 3:**
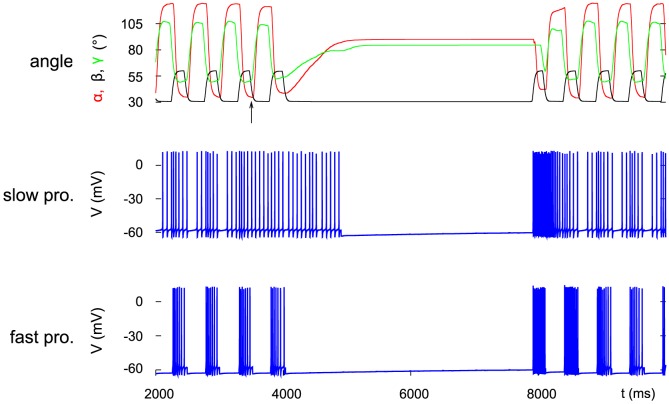
Simulation results illustrating the stop and start of stepping in the model. Top panel: time evolution of the three joint angles 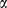 (retraction, red), 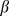 (levation, black), and 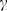 (flexion, green). Middle and bottom panel: activity of the slow and fast protractor motoneuron, respectively. The arrow in the top panel indicates the onset of the central stop command. The ground contact is, as during stepping, established by activation of the depressor muscle at the end of the swing phase, and the steady state is attained in the subsequent stance phase. Note that the activity of the fast protractor motoneuron is stopped only at the end of the last protraction (swing) phase but the slow one has a prolonged and enhanced tonic activity that lasts until the steady-state 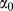 of the angle 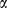 is reached. At start, as shown in the top panel, the levator muscle is activated first initiating a swing phase (protraction and extension, respectively, in the other two neuro-muscular systems). The motoneurons start with a high firing frequency (middle and bottom panel).

Fig. 3 illustrates the result of these interacting processes. The top panel shows the three angular movements 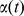 (red), 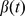 (black), and 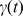 (green), and their phase relations. The arrow indicates the central stop command. As it arrives at the start of a retraction (stance) phase, this phase of the stepping cycle is completed before the stopping process becomes effective at the beginning of the next protraction (swing) phase. Then a permanent ground contact is established (black trace). Finally, the complete steady state is preceded by a retraction (stance) phase. The middle and the bottom panel display intracellular activity of the slow and the fast protractor MNs, respectively. Note that whereas the fast MNs are rhythmically active, the slow ones show tonic activity, especially after the inhibition of the fast MNs. This happens, because we additionally assumed that, in the model, the central stop command also enhanced the activity of the slow protractor, rectractor, extensor and flexor MNs (via inhibition of their inhibitory INs by increasing the corresponding 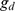 values, see eqn 8 above and Fig. 1). There is only weak indirect experimental evidence for the prolonged tonic activity of the slow MNs. Records in [10] show tonic activity of the slow extensor MN (SETi) in the stick insect and in the locust in the absence of the fast MN activity at fixed middle leg, that is a prolonged tonic activity of the slow MNs can be seen.

**Figure 4:**
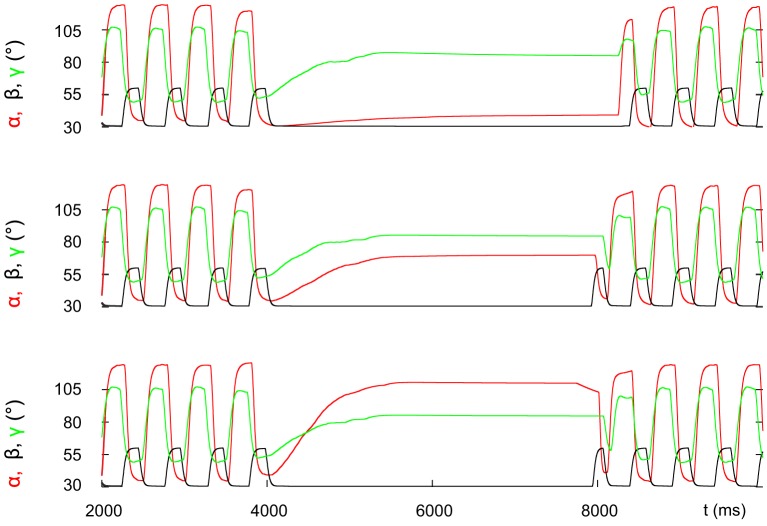
Steady state at three different angles 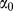 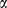: red trace. Top panel: 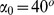, middle panel: 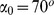, bottom panel: 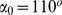. The steady-state values of 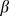 and 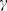 are always the same (cf. text). The central stop command occurs at the same time as in Fig. 3. The stepping starts with a swing phase when 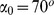 (middle panel) and when 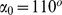 (bottom panel) like in Fig. 3 but when 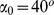, the stepping commences with a stance phase (top panel). See subsection Start of stepping.

The process of stopping described above in detail is independent of the steady-state angle 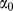 to which 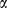 converges. Fig. 4 demonstrates this result. The different steady-state values of 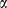 were set by making use of the recruitment property of the model (cf. Methods and [34]). The steady-state values of 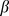 (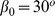) and 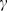 (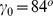) were always the same since the projection of the standing position into the vertical plane is unchanged in all cases (cf. Fig. 2). The cases depicted in Fig. 4 do have experimental correlates. In [1], they correspond well to the steady-state horizontal position of the femur of the front, middle and hind leg, respectively (cf. [1], Fig. 2). Moreover, some unpublished observations (Grabowska, unpublished data) also appear to be in agreement with our simulation results.

**Figure 5:**
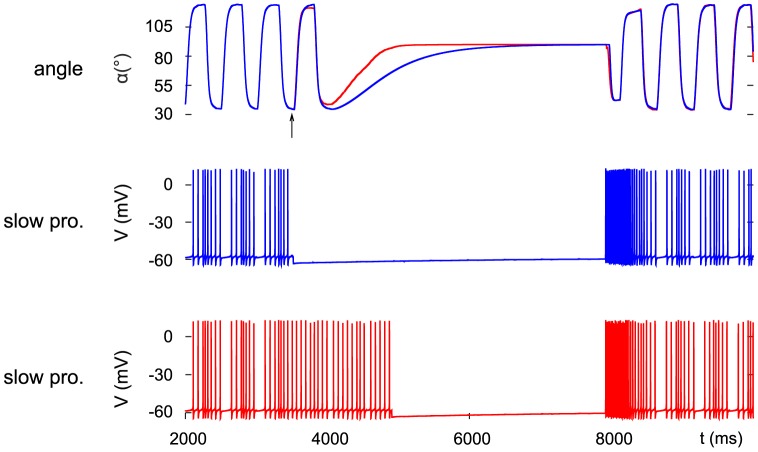
The role of the prolonged activity of the slow protractor and retractor motoneurons and muscle fibres. Top panel: time evolution of the retraction angle 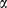; middle panel: activity of the protractor (and retractor motoneuron – not shown) is stopped at the occurrence of the central stop command; and bottom panel: prolonged and enhanced activity of the protractor (and retractor motoneuron – not shown). In the top panel, the colour of angular movements corresponds to that of the motoneuron activity in the two other panels. Note that prolonged and enhanced activity of the slow motoneurons (bottom panel) results in an accelerated convergence of 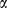 to its steady-state value (red curve, top panel).

Fig. 5 demonstrates an important putative physiological role, as mimicked by the model, of the slow muscle fibres and of the slow MNs innervating them during the stopping process. It shows that the convergence of the angle 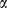 to its steady-state value is markedly faster when the slow protractor and retractor MNs exhibit prolonged and enhanced tonic firing activity (compare the corresponding angular movements: red, blue in Fig. 5). Although this result is shown here for the protractor-retractor neuro-muscular system only, it is also valid for the extensor-flexor neuro-muscular system. That is, the slow MNs of the latter system work according to the same time schedule during the stopping process as their counterparts in the protractor-retractor system. However, the situation is different in the levator-depressor neuro-muscular system. We found in the simulations that when the slow MNs of this system were tonically firing, a stable and permanent ground contact in the stance phases could not be produced because of the tonic firing of these very MNs. Hence, we attributed the slow MNs in the levator-depressor system the same rhythmic activity as that of the fast MNs, i.e we made no difference between the activities of the slow and fast MNs.

**Figure 6:**
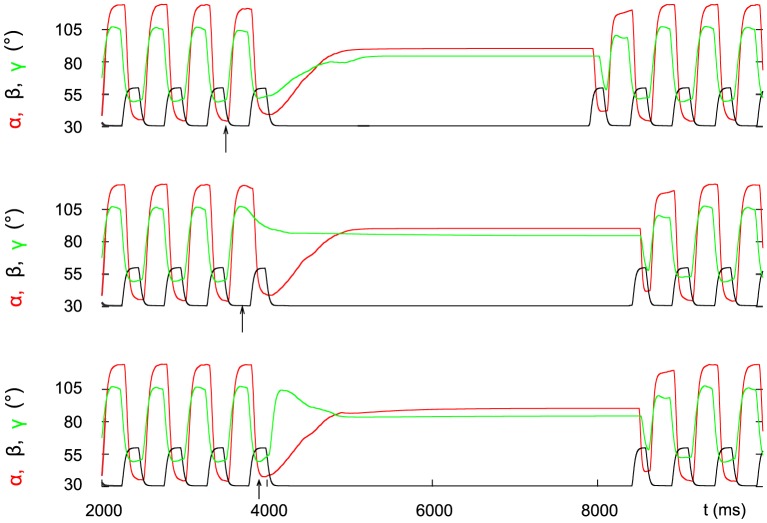
Central stop command evoked in three different phases of the stepping cycle. Top panel: at the start of the retraction phase (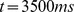); middle panel: at the end of the retraction phase (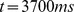); bottom panel: towards the end of the protraction phase (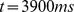). Note that the qualitative behaviour of the model remains the same in all three cases both at stop and start, except for the extensor-flexor system which occasionally shows an extra (partial) extension (bottom panel). For further explanation, see text.

Finally, we show in Fig. 6 that the behaviour of the system remains basically unaffected by the time of onset of the central stop command. In the simulations, we chose a number of different occurrence times of the central stop command that fell within the same stepping cycle (of 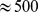 ms), i.e. occurred at different phases of that cycle, in each case. Every time, the levator-depressor system behaved exactly the same way, whereas the protractor-retractor system showed qualitatively the same behaviour by performing a retraction (in the stance phase) before reaching its stationary position (the angle 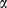 approaching its steady-state value 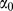 from below). The extensor-flexor system, however, did not always exhibit exactly the same behaviour. Occasionally, as shown in the bottom panel of Fig. 6 (green trace), it carried out first a flexion followed by a partial extension during which the angle 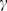 monotonically converged to its steady-state value. This happened because the central stop command arrived just when the flexion started. Note that the extension during stepping finishes a bit earlier than the corresponding protraction (Fig. 6), like in the stick insect [15]. This is why the protractor-retractor system is still in the protraction phase when the extensor-flexor system has already completed its extension phase.

### Start of stepping

Theoretically, there are two possible ways of starting from a standing, stationary position: performing first a swing phase (protraction) or a stance phase (retraction). We implemented both possibilities in the model. Indeed, the stick insect seems to make use of both of these possibilities [1] and (Grabowska, unpublished data). Whether a swing phase or a stance phase would be produced first by a (middle) leg depends on the actual steady-state value of 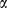 in the standing position. In the model, we defined a critical angle that separates these two cases. We set its value to be 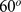. In the extensor-flexor system of the stick insect, it was found that the relative frequency of the middle leg starting with a swing or a stance phase monotonically depended on the angle 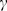 [23]. Our assumption of a critical 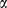 angle seems therefore reasonable, even though the choice of the specific value of 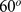 remains somewhat arbitrary.

The simulations show examples for both cases. In Fig. 4, top panel in which 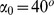, the protractor-retractor system starts with a retraction, accompanied by a flexion in the extensor-flexor system, while the leg has still ground contact (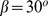), i.e. the leg is in the stance phase. Only when both retraction and flexion are completed will the levator-depressor system be enabled to lift the leg off the ground. This appears to be in good agreement with findings in [1] and (Grabowska, unpublished data). In the model, the stepping starts at a well-defined phase of the activity of the protractor-retractor CPG (C1 and C2 in Fig. 1): when the “retractor” CPG neuron (C1 in Fig. 1) is about to reach its maximal depolarization by exceeding a threshold value 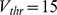 mV. Note that all three CPGs are active and synchronized, because the load and position sensory signals represented by the angle 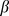 are active, i.e. 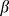 is below its critical value (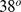 for the protractor-retractor system, and 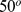 for the extensor-flexor system; cf. Fig. 1 and Methods). Summarizing the starting conditions in this case in form of equations, we have
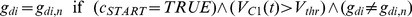
(14)

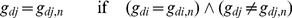
(15)

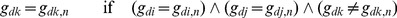
(16)for 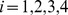, 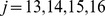, and 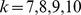. The boolean variable 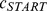 represents the central start command. These eqns also show that after the protractor-retractor system is activated, in a retraction (stance) phase, the extensor-flexor neuro-muscular system is activated next with a flexion, provided that the protractor-retraction system is already active. The levator-depressor neuro-muscular system is finally activated, once both of the other systems are already active. Because of the synchronization between the three CPGs, the leg's next lift-off will only occur when the retraction and the flexion have been completed. Note that in all three systems the slow and fast MNs are activated at the same time.

In the two other panels of Fig. 4 in which 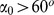, the stepping starts with lifting the leg off the ground. This kind of start was also observed in the animal [1] and (Grabowska, unpublished data). In the model, the mechanical movement is triggered when the “levator” CPG neuron (C3 in Fig. 1) reaches the depolarization level of 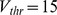 mV. The corresponding equation reads
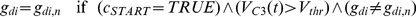
(17)for 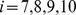. Then the angle 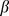 increases by activation of the levator muscles in the levator-depressor system. The levation induces protraction and extension, respectively, in the two other neuro-muscular systems via the load and position sensory signals represented by the angle 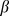 (Fig. 1). Here, too, in all neuro-muscular systems, the slow MNs and muscles are simultaneously activated with the fast ones. Note that the extension starts later than the protraction due to the different thresholds of 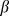 in the two systems (cf. Methods and [13]).

## Discussion

In this paper, we set out to provide an explanation of how locomotion in the stick insect is stopped and started by making use of a suitable neuro-mechanical model. This model is based on our one in the accompanying paper [34]. We complemented it by making physiologically plausible assumptions with regard to the stopping and starting of stepping. Among these assumption two are of fundamental importance. The first one is on the coordinating roles of sensory signals reflecting position and velocity of the leg's main joints. The existence of such sensory signals has been well established in experiments [7]–[9]. Our assumption, more precisely a set of assumptions, relates to their crucial role in coordinating the activities of the leg's neuro-muscular systems during locomotion, standing, and during the transition processes between these states. These sensory signals can thus trigger sub-processes in the same or in a different neuro-muscular system. For example, the negative value of the angular velocity 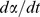 results in the inhibition of the fast MNs in the protractor-retractor neuro-muscular system at stopping; the activity of the levator-depressor can only be blocked at stopping, once the fast MNs of both the protractor-retractor and the extensor-flexor system have been inhibited. A model not incorporating the specific set of assumptions we used here would have to have quite different properties and mechanisms of intra-leg coordination. The second fundamental assumption was not made explicitly, since we used it in our earlier work [13], [14]. Its main claim is that neither stopping nor starting of stepping requires any direct interference with the activity of any of the three CPGs. As it should be clear from the Results, and the eqns describing the actions of the sensory signals, the driving currents (the conductances 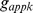) to the CPGs were at no point changed. That means that the CPGs ran autonomously all the time without direct interference. All actions on the MNs, hence on muscles, were conveyed via the inhibitory INs connecting the CPGs to the MNs. Thus all transient signals that would arise by directly changing the activity of the CPGs (i.e. changing the conductances 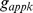) could be avoided. Equipped with this property, our model is capable of responding to unexpected changes (e.g. sudden stop or start) much faster than models in which the activity of the CPGs is directly manipulated. Quite recently, experimental evidence has arisen that the CPG is not affected during change in the MN activity. It has been shown by Rosenbaum et al. (unpublished results) that the activity of the protractor-retractor CPG remains unaffected during a switch from forward to backward walking of the stick insect when, however, the protractor and retractor muscles, hence MNs, exchange roles. We also successfully modelled this phenomenon the same way, i.e. without changing the activity of the protractor-retractor CPG in the model [14].

We also assigned the slow muscle fibres residual stiffness and took the effects of the common inhibitory MNs on the function of slow muscle fibres into account [17]–[19], [21]. Having incorporated these properties into the new version of the model, we could mimic both stopping and starting of locomotion (stepping). It turned out that both processes, though basic elements of locomotion, required precise coordination of the three main neuro-muscular systems of a leg. The simulations with the model highlighted the details of the coordination between those neuro-muscular systems. The constituent processes are physiologically plausible. They are i) sensory signals representing position (angle) or (angular) velocity at the individual joints as well as load signals, represented by the angle 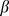); ii) neuronal signals driving the MNs of the system; iii) activities of the MNs driving the fast or slow muscles they innervate; and iv) contractions of the slow and fast muscles producing the mechanical movement of the individual leg joints. Gradual muscle recruitment in the model ensures that any stationary position (angle 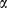) of the femur can be attained. The steady state, posture, is maintained in the model by the residual stiffness of the slow muscle fibres, in agreement with experimental findings [16], [17]. This property of the model does not contradict the result in the accompanying paper [34] that steady state is maintained by enhanced tonic firing of the slow MNs, hence co-contractions of the slow muscle fibres in the protractor-retractor system. There, the slow muscle fibres did not possess any residual stiffness, and the co-contraction of the slow muscle fibres was thus required to maintain a stable steady state. In reality, both possibilities are likely to exist and co-exist [10], [16].

There are important experimental results that underpin the assumption made when constructing the model. First of all, it is well established that slow and fast muscle fibres have their dedicated MNs driving them, and that the fast muscle fibres do not contribute to maintaining the steady state (posture) in insects [10], [11]. It has also been experimentally confirmed that there are position-, velocity- and load-dependent sensory signals in the animals [2], [7]–[9]. The existence of residual stiffness in the slow muscle fibres, and the related function of the common inhibitory MNs (CI1-CI3) to remove it, was demonstrated [10], [11], [17]–[19], [21]. It was also observed that stop of stepping does not occur randomly during a step cycle, and a leg grinds to a halt when it has ground contact, i.e. in the stance phase [1]. The model presented in this paper incorporates all these properties, and, as far as we are aware of, it is the first one to do so.

The model behaviour is in good agreement with the experimental findings. In the simulations, the time evolution of the angles at the leg joints produced coordinated movement of the whole leg both at stop and start of stepping, similar to the observed ones in the stick insect [1] (Grabowska, unpublished data). In particular, our model is capable of reproducing the preferred steady-state positions of the front, middle and hind leg in the stick insect: the front leg (femur) being near to its anterior extreme position, the hind leg near to its posterior extreme position, and the middle leg somewhere between them. (Compare the three panels in our Fig. 4 with Fig. 2 in [1].) The fast MNs fire rhythmical bursts during locomotion due to their rhythmic inhibition by the associated CPG [24]. The slow MNs, by contrast, produce tonic firing, at least in the case of the extensor muscle [10]. In the model, we applied these firing modes both in the protractor-retractor and the extensor-flexor system. In the levator-depressor system, however, we found that tonic firing of the slow MNs resulted in incomplete ground contact in the stance phases. In the levator-depressor system, we therefore implemented identical rhythmic firing of both the fast and the slow MNs. The experimental evidence, we could find is inconclusive as to the activity of the slow MNs in the levator-depressor system [25], [26]. Hence this part of the model remains highly hypothetical. However, we succeeded in identifying a putative physiological role of the slow muscle fibres at stopping. The prolonged and enhanced tonic activity of the slow protractor and retractor MNs driving the corresponding slow muscle fibres substantially accelerated convergence of the angle 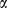 to its stationary value (Fig. 5). The same is true for the slow MNs and muscle fibres of the extensor-flexor system. This is a hitherto unidentified function of the slow MNs and muscle fibres in both systems, and it is thus an important prediction of our model. It should be mentioned here that there is some uncertainty in the physiological interpretation of the apparent “stop” and “start” commands in the model. Currently, there is no experimental way to find out whether such single command signals are produced by the brain, and if so when and in which part of the brain they exactly arise.

As pointed out in the Methods and Results, the sensory signals representing position (angle) and (angular) velocity (e.g. 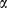, 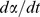) were not implemented in the model as neuronal signals but only formally, by computing their effects on the neuronal and muscular activity in an abstract way. This is also true for the role of the common inhibitor MNs CI1-CI3. Their effect: removing the residual stiffness of the slow muscle fibres during locomotion and restoring it at stopping was implemented only, as an abstract mathematical function obeying logical conditions.

### Physiological viability of the model

**Figure 7:**
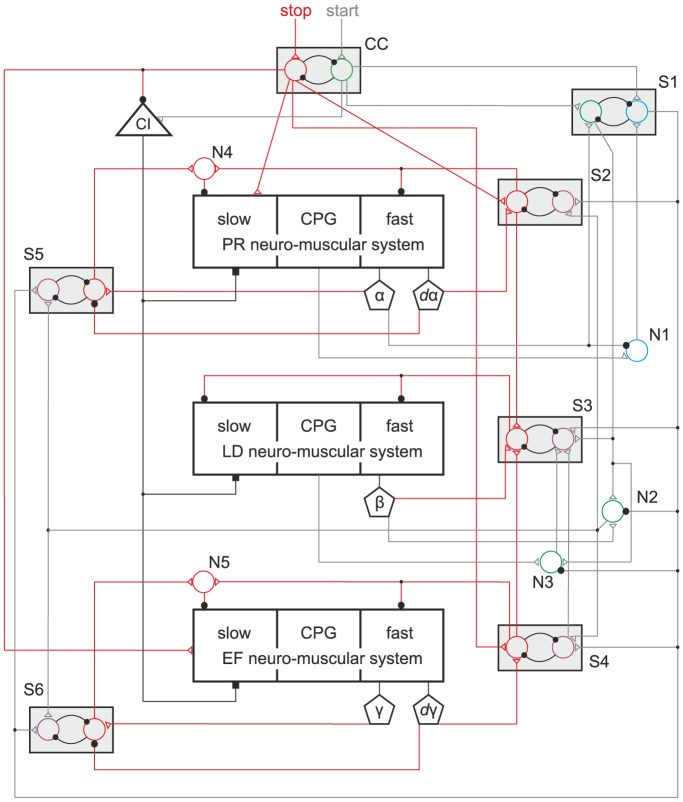
The physiological viability of the model. Active pathways at stopping. The three large boxes in the centre of the figure are the three neuro-muscular systems as indicated from Fig. 1. Their existence is physiologically well established [15], [27]. Their inner structure has been partially confirmed in experiments [10], [11], [24], [27]–[31]. The small boxes CC and S1–S6 are CPG-like functional units consisting of mutually inhibitory neurons. Such small networks of mutually inhibitory neurons do exist in the nervous system of athropods [32] and other animals [33]. However, such units with the specific roles assigned to them in the present model have not been identified. Triangle CI represents the common inhibitory motoneuron. Its existence is proven in experiments [17]–[19], [21]. N1-N5 are neurons whose existence with the specific function they have in the present model has not yet been established. Pentagons 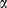, 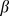, 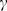 are sense organs where signals reflecting the position (angle) are generated; pentagons 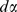, 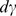 are sense organs where signals reflecting the (angular) velocity are generated. Such sense organs do exist in insects (e.g. [6]–[8]). Small empty triangle on neurons or on system boxes are excitatory synapses. Filled black squares symbolize inhibition by CI of the slow muscle fibres. Filled black circles are inhibitory synapses. Very small black circles along paths (neuronal connections) are branching points. Red pathways are the connections that are active at stop of stepping. Grey pathways are the connections that are active at start of stepping (see. Figs. 8–9). For more explanation as to the workings of this network, see text.

**Figure 8:**
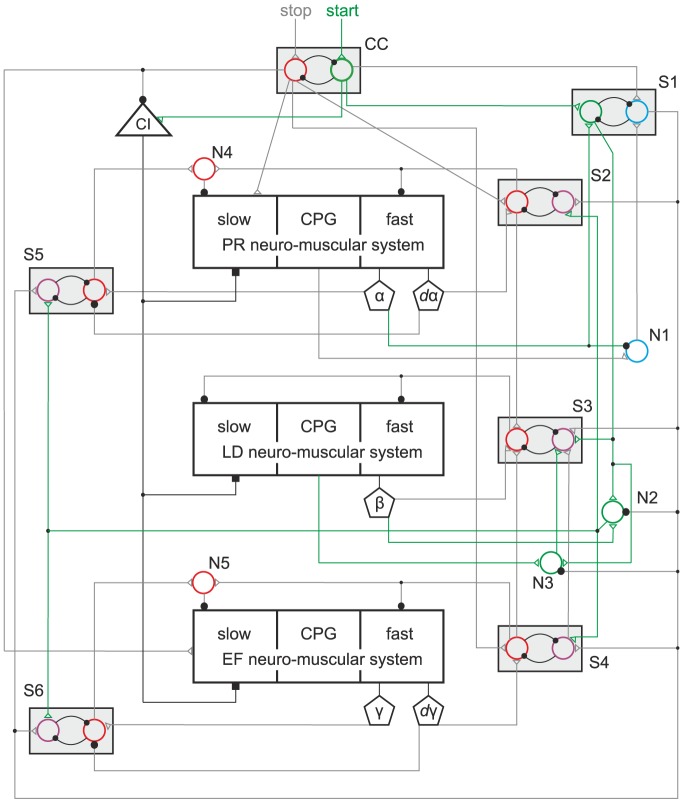
The physiological viability of the model. Active pathways at start. The same network as in Fig. 7: Here, the pathways that are active when the starting process commences with a protraction (swing phase) are highlighted in green. The other pathways are suppressed by having been drawn in grey. For explanation of how the network works in this case, see text.

**Figure 9:**
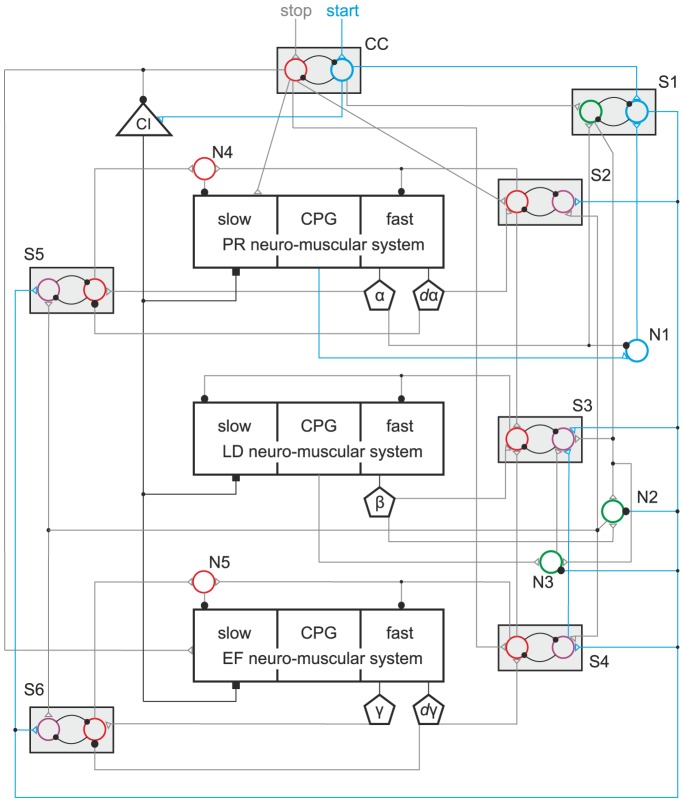
The physiological viability of the model. Active pathways at start. The same network as in Figs 7–8: Here, the pathways that are active when the starting processes commences with a retraction (stance phase) are highlighted in blue. The other pathways are suppressed by having been drawn in grey. For explanation of how the network works in tin this case, see text.

Now, we show that our model is, at least in qualitative terms, physiologically viable. We present, in Figs. 7–9, a neuronal network that could be regarded as a physiologically meaningful implementation of our model. The network structure and its elements are not entirely hypothetical. The existence of the three main building blocks (neuro-muscular systems) has reliably been established in experiments [15], [27]. Basic properties of the local networks of the neuro-muscular systems could also be gleaned from experimental data [10], [11], [24], [27]–[31]. In addition, it is known that pairs of mutually inhibitory (nonspiking) neurons or groups of neurons capable of exhibiting bistability do exist in the nervous system of athropods [32] and other animals [33]. All these facts lend the network in Fig. 7 a sufficient physiological basis.

We did not implement this network as a quantitative model to be used in simulations, since there are too many unknown parameter values, especially synaptic weights, in it, hence the attribute “qualitative”. Nevertheless, we believe that presenting it does make a contribution to bringing an abstract model and a physiological system closer to each other, and to furthering a better understanding of how the model works.

Here we give an account of how the network works at stop and start of locomotion (stepping), respectively. The stop is initiated by a central stop command: red path labelled with “stop” into unit CC. When the stop command arrives CC, it inhibits its alternative, the “start”, command (green neuron). The stop command enhances the activity of the slow MNs in the protractor-retractor and the extensor-flexor system (see red pathways starting at the red neuron in unit CC and ending in red empty triangles on the boxes representing these two systems). The fast MNs of the protractor-retractor system are inhibited, the activation of the fast muscle fibres innervated by them stopped, as soon as 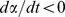 (cf. Results), because the red neuron in S2 is activated and sends an inhibitory signal to the MNs of the fast protractor-retractor system. It also inhibits the other (magenta) neuron in S2. An analogous cascade of events takes place in the extensor-flexor system (unit S4). Now, if the red neurons in the units S2 and S4 are excited, they send a signal each to their red counterpart in unit S3. In order to enter the excited state, the red neuron in S3 also needs an excitation from the sense organ sensing position (angle 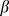) in the levator-depressor system. This sensory signal is evoked, if 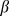 is in a close neighbourhood of its steady-state value (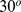). Then the red neuron in S3 is activated and inhibits both the fast and slow MNs of the levator-depressor system. By this, a permanent ground contact of the leg is established. Note that the slow MNs in both the protractor-retractor and the extensor-flexor system are still active. Moreover, their activity has been boosted by the stop command. The slow MNs of the protractor-retractor system are inhibited as soon as the red neuron in S5 is activated. For that to happen, it is required that the angle 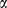 be close to its steady-state value, and that 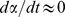. If this is the case, the red neuron in S5 will receive an excitatory signal originating in the sense organ that signals the value of 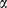, and the inhibition coming from the sense organ signalling the value of 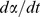 will vanish. Thus the red neuron in S5 becomes excited and sends an excitatory signal to neuron N4, which has already been receiving another excitatory signal from S2. The latter has, on its own, been insufficient to activate neuron N4. Now, it is activated and it inhibits the slow MNs of the protractor-retractor system. The mechanical movement of the femur grinds to a halt. The events in the extensor-flexor system are completely analogous. By inhibiting all MNs in each of the three systems, the leg stops moving. The central stop command inhibits the common inhibitor CI directly, and the excitatory signal to it from unit CC also ceases. This abolishes the inhibition by CI of the slow muscle fibres in all muscles. That is, residual stiffness in the slow muscle fibres becomes active, and it determines the stationary position of the leg. Of course, as soon as the red neurons of the units S2–S6 (and CC) become activated, they inhibit their magenta counterparts in the same unit strongly enough to prevent any signal flow through them.

The start of stepping can occur in the model in two different ways: the leg starting with a swing phase or a stance phase. The steady-state value 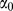 of the angle 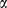 determines which of the two cases will occur. In any case, the foremost action is that the central start command activates the ‘start’ neuron (green in Fig. 8 and blue in Fig. 9) in unit CC, hence inhibits the ‘stop’ (red) one whose excitatory output immediately ceases. Let us consider first the case when 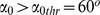. That is, the stepping will start with a swing phase. For this variant, the green pathways in Fig. 8 will be used. Because of the condition for 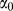 above, the position signal for 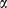 (cf. Fig. 8) excites the green neuron in unit S1, together with the start signal, and inhibits neuron N1. In turn, the blue neuron in S1 is inhibited, and, as a consequence, all blue pathways will be blocked (Fig. 9). The green neuron in S1 then excites the neurons N2 and N3 enabling the excitatory pathways through them to become active. The pathway through N3 from the CPG conveys an excitatory signal to the magenta neuron in S3 when the CPG of the levator-depressor system starts the levation phase. This excitatory signal and the start signal conveyed by the green neuron in unit S1 activate the magenta neuron in unit S3, which inhibits the red neuron in the same unit. This, in turn, abolishes the inhibition of both the fast and slow MNs of the levator-depressor system. Since the CPG is in the levation phase, the angle 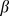 increases, i.e. the leg is lifted off the ground. To transmit this information to the other neuro-muscular systems, an excitatory signal is sent via neuron N2 to the magenta neurons in the units S2, S4, and S5, S6. The magenta neurons in these units become activated, hence their counterparts (the red neurons) in the same unit inhibited. Accordingly, the inhibitory signals to the fast MNs disappear directly, whereas the inhibition to the slow MNs indirectly by deactivation of the neurons N4 and N5, respectively. By virtue of the sensory signals represented by the angle 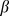 (cf. Fig. 1), the CPGs of the protractor-retractor system and the extensor-flexor system are synchronized to that of the levator depressor system [13], [14], [34]. The stepping therefore starts with a swing phase (see, for example, Fig. 3 or Fig. 6).

The alternative to this process is one with an initial stance phase. An example is displayed in the top panel of Fig. 4. This process uses the blue pathways in Fig. 9. In this case, the retractor phase of the CPG of the protractor-retractor system triggers the start of the stepping. Since there is now no 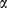-signal to S1 and to neuron N1, the latter is activated, and it activates, together with the central start command, the blue neuron in unit S1. This neuron, in turn, inhibits the neurons N2 and N3, hence no excitatory signal from the 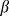-sensor and the CPG of the levator-depressor system can reach the magenta neurons in units S2 and S4. (The 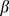-signal to unit S3 is irrelevant in this case, because it is present only at the tarsus reaching the ground.) Instead, excitatory signals from S1 arrive these (magenta) neurons, activate them and inhibit the corresponding red neurons simultaneously. This is also true for units S5 and S6, which control the slow MNs in the protractor-retractor and the extensor-flexor system (cf. direct blue pathways to these units from unit S1). The red neurons will also be inhibited in units S5 and S6. This means that the inhibition of both the fast and the slow MNs will be abolished in both the protractor-retractor and the extensor-flexor neuro-muscular system. Since the levator-depressor system is still inhibited, ground contact is maintained, and because the protractor-retractor CPG is in the retractor phase, a stance phase of the stepping cycle ensues. The magenta neuron in unit S3 is activated by simultaneous excitatory signals from S1 and S4, the latter belonging to the extensor-flexor system. The red neuron in S3 is now inhibited, and the inhibition of the fast and slow MNs of the levator-depressor system is abolished. Thus the next swing phase can commence when the CPG of the levator-depressor system reaches the next levation phase of the stepping cycle. Irrespective of whether the stepping starts with a swing or a stance phase, the start signal immediately activates the common inhibitor MN (CI in Figs. 8–9) by inhibiting the red neuron in CC, which has an inhibitory effect on CI and, at the same time sending a permanent excitatory signal to CI (Figs. 8–9).

## Conclusions

Having shown that our model is physiologically viable, i.e. it can, at least qualitatively, be implemented by using a neuronal network with physiologically realistic neurons and synapses, we should like to argue that it is physiologically relevant, too. First, it was constructed by using experimental results and physiologically reasonable assumptions. The signals in it are of neuronal origin. Thus there exists a close correspondence between the model and its biological counterpart at several levels of complexity. This makes the interpretation of the simulation results easier and more plausible. Second, our model, in contrast to earlier ones, allows functional differentiation between static and dynamic aspects of movement control. Third, even though our model has been constructed by using experimental findings from the stick insect, the main result achieved with it, namely showing of how intra-leg coordination is organized during stop and start of locomotion (stepping) may be generalized to elucidate analogous processes in other insect species, too. We even venture to suggest that some details of the model could perhaps be used in constructing insect-like robots. In this sense our model might attain a more general relevance and, maybe, significance than just relating to physiological processes in the stick insect.
